# Prevalence of Intestinal Parasite Infections and Their Associated Factors among Food Handlers Working in Selected Catering Establishments from Bule Hora, Ethiopia

**DOI:** 10.1155/2021/6669742

**Published:** 2021-08-20

**Authors:** Sunil Tulshiram Hajare, Robe Kuti Gobena, Nitin Mahendra Chauhan, Feleke Eriso

**Affiliations:** Department of Biology, College of Natural and Computational Sciences, Dilla University, Dilla, 419 SNNPR, Ethiopia

## Abstract

Intestinal parasites are responsible for one of the major health problems like food contamination with socioeconomic effects in the world with a prevalence rate of 30-60%, in developing countries that lie within tropical and subtropical areas. They pose a reasonable public health burden, particularly in low- and middle-income countries, including Ethiopia. Globally, due to intestinal parasitic infections, around 3.5 billion people are affected and more than 200,000 deaths are reported annually. Around 50000 deaths yearly are caused by intestinal parasites in Ethiopia. As such, intestinal parasites perceived global and local burdens to various countries. The risk of food contamination depends largely on the health status of the food handlers, their hygiene, knowledge, and practice of food hygiene. Food handlers with poor personal hygiene and sanitation conditions are the major potential sources of intestinal helminthes and protozoa worldwide. The proposed study was aimed at evaluating prevalence of intestinal parasitic infections and their associated factors among food handlers working in selected catering establishments. A cross-sectional study was conducted in Bule Hora Town from March to April 2020. A total of 136 catering establishments were selected using a systematic sampling technique. Data analysis was done using SPSS version 20. The prevalence of intestinal parasites in this study was 46.3%. *Entamoeba histolytica* was the most predominant parasite (33.3%, i.e., 21/63) while *Giardia lamblia* was the least (11.1%, i.e., 7/63). Consumption of vended or borehole water and hygienic practices such as hand washing before eating, after using toilet, before cooking and trimming of finger nail and wearing proper working clothes and shoes were statistically significant with intestinal parasitic infection (*P* < 0.05). Generally, the prevalence of intestinal parasitic infection in this study was high and contributed by low socioeconomic status and poor environmental and personal hygiene. Measures including education on personal hygiene, environmental sanitation, drinking water supply, regular medical checkups, and treatment should be taken into account to reduce the prevalence of intestinal parasites.

## 1. Introduction

Infections caused by intestinal parasites are widespread causing significant problems in individuals and public health, particularly in developing countries, with a prevalence rate of 30-60.0% [1]. In addition, these parasites are responsible for one of the major health problems with socioeconomic effects especially, in developing countries within tropical and subtropical areas [2]. Rural-to-urban migration rapidly enhances the number of food eating places in towns and their environs. Some of these eating establishments have poor sanitation and are overcrowded, facilitating disease transmission, especially through food handling [3].

Globally, due to intestinal parasitic infections, some 3.5 billion people are affected; 450 million are symptomatic, and yearly more than 200,000 deaths are reported [2]. A study conducted in Riyadh, Saudi Arabia, testing for parasitic infections among food handlers showed that 12.8% of the specimens tested positive for the parasites [2, 4]. A similar study conducted in the city of Makkah during Hajj season investigating intestinal parasitic infection among food handlers detected 31.9% of the food handlers [2].

Reports indicate that food handlers working in hotels, hostel mess, and other catering services reported personal hygiene and sanitation conditions which are the major potential sources of intestinal helminthes and protozoa from many developed and developing countries all over the world [5–8]. Intestinal parasites are transmitted either directly or indirectly through food, water, or hands highlighting the importance of fecal-oral and human-to-human transmission [7–9]. Asymptomatic carriers, in particular, are a public health hazard, especially if they work as food handlers where they may become a source of intestinal parasitic infection to others [4]. The parasites are not easily detected when they get into the human body; hence, they can live in the human body for long without being diagnosed. They are responsible for major health problems with socioeconomic effects in the world and especially so in developing countries in tropical and subtropical areas [10].

The helminthes *Taenia saginata*, *Hymenolepis nana*, *Ascaris lumbricoides*, *Strongyloides stercoralis*, *Trichuris trichiura*, and *Enterobius vermicularis* and hookworms predominantly *Necator americanus* and *Ancylostoma duodenale* as well as the protozoans mainly *Giardia lamblia* and *Entamoeba histolytica* are the major intestinal parasites leading to digestive disorders [11]. According to WHO [12] every year, 45,000 deaths are directly attributed to hookworm infections, and another 4300 to *Ascaris lumbricoides* (roundworm). *Entamoeba histolytica*, which causes amoebiasis, is estimated to cause severe disease in 48 million people, killing 54,000 each year. Multiple infections with several parasites (e.g., hookworms, roundworms, and amoebae) are common, and their harmful effects are often aggravated by coexistent malnutrition or micronutrient deficiencies [13].

In Africa, particularly sub-Saharan Africa, a study carried out over a ten-year period between 1999 and 2009 reported a 30.2%-55.6% prevalence of intestinal parasites among the vast majority of the people [14]. With the rapid increase in urbanization, industrialization, and tourism, food and drinking establishments are gaining popular in both industrialized and developing countries. In Ethiopia, the presence of intestinal parasitic infections has been reported to cause close to 50,000 deaths annually mainly due to the low standards of hygiene in the country, like any other developing country. This is mainly due to poor hygienic food handling and preparation practices particularly in public food establishments [15]. Strengthening the above fact, different studies conducted in different parts of Ethiopian towns revealed that there were poor personal hygienic practices, inadequate sanitary facility, improper handling and storage of food and food utensils, and improper waste storage and disposal. A significant difference in the number of trained and untrained food handlers with regard to practices of hand washing and sink accessibility was related to hand washing, which suggests that sink accessibility promotes hand washing [15, 16]. Health and hygiene of food workers are major determinant factors for food safety [17–20].

The spread of disease by food handlers is a common and persistent problem globally [21]. Food handlers with poor personal hygiene working in the foodservice settings can be infected by different enteropathogens [22], where they can cause fecal contamination of foods by their hands during food preparation and which may be transmitted to the public [21]. Therefore, a proper screening procedure for food handlers is helpful in the prevention of probable morbidity and the protection of consumer health. The risk of food contamination, therefore, depends largely on the health status of the food handlers, their hygiene, knowledge, and practice of food hygiene [23]. In the proposed study area, there are 408 public catering establishments regardless of licensing status. In most cases, establishments give attention only to the availability and service of food, but not on its safety and quality. Furthermore, data regarding the food handler's health status before and after employment and training certificate are scarce. As a result, consumers may easily be threatened by food-borne diseases of the enteric pathogens and other disease-causing agents contaminating the food.

Unfortunately, data is lacking on the burden of intestinal parasites among those eating and drinking establishments from Bule Hora Town, Ethiopia. Therefore, the proposed work was a first attempt to determine the prevalence and risk factors associated with the food handlers among selected places from the studied area.

## 2. Materials and Methods

### 2.1. Description of the Study Area

**Figure 1:**
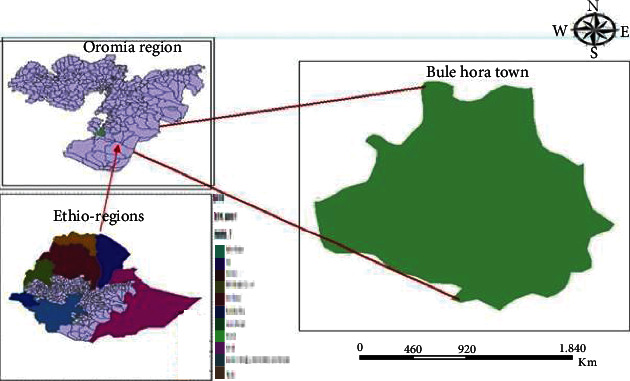
Map of the study location of Bule Hora Town, Ethiopia.

Bule Hora Town is a town situated in the southern part of Ethiopia. It is located on the paved Addis Ababa-Moyale highway in the West Guji Zone of the Oromia region. It has a latitude and longitude of 5°35′N and 38°15′E, respectively, and an altitude of 1716 meters above sea level (Figure 1). The 2014 national census reported a total population of 27,820 for Bule Hora Town, of whom 14,519 were men and 13,301 were women. 6,507 households and 6,246 housing units were counted. The current population density is estimated at 3,079 people per square kilometre. 75% of the population has access to municipal water while the remaining 25% used wells, springs, and other sources. The town has a 408 registered catering establishment regardless of licensing status [24, 25].

### 2.2. Design of Study and Settings

A descriptive cross-sectional study was conducted between March and April 2020, and both quantitative and qualitative approaches were preferred to gather desirable information. The data was gathered from a pool of participants with varied characteristics and demographics known as variables. It contains multiple variables at the time of the data snapshot.

#### 2.2.1. Study Population

The study population includes all catering establishments like hotels, bar and restaurants, cafe and restaurants, dining rooms, cafes, food and groceries, cafeteria found in Bule Hora Town, and all persons employed and working as food handlers in the above selected catering establishments.

#### 2.2.2. Sample Size Determination

The formula by Yamane [26] was used to calculate the minimum sample size required to achieve a 95% of power as shown below:

(1) $$\begin{array}{c} n=\frac{N}{1+N}\left(e^{2}\right), \end{array}$$

where *N* is the population size and *e* is the level of precision. Therefore, from a preliminary study, we have 408 catering establishments in Bule Hora Town. Hence, the size of our sample was calculated as follows:

(2) $$\begin{array}{c} n=\frac{408}{1+408}\left(0.052\right). \end{array}$$

Assuming 1 + 408 (0.05^2^) is equal to 3; therefore,

(3) $$\begin{array}{c} n=\frac{408}{3}=136. \end{array}$$

#### 2.2.3. Inclusion Criteria

The study was consented and enrolled those working in catering establishment found within Bule Hora Town, willing to participate and provide a stool sample as well as undergo a 30-minute face-to-face interview.

#### 2.2.4. Exclusion Criteria

The participants were excluded from the study if they are working outside of catering establishment from Bule Hora Town and unwilling to participate in accordance with the written consent to participate and were not ready to give a stool sample and undergo a 30-minute face-to-face interview.

### 2.3. Study Procedure

A structured and semistructured questionnaire was preferred to collect data on sociodemographic, economic, personal, and environmental characteristics. The questionnaire was first prepared in English and then translated into the local language. After data collection, the data collected in the local language was retranslated into English by taking the help of people with expertise to assure consistency.

### 2.4. Sampling Procedure

All catering establishments employed workers (whether on a temporary or permanent basis) who handle the food, which were constituted in the sampling frame. For catering establishments, the census was done first to get a list of each different type of catering establishments from a selected place, which was found to be 408 in total. Finally, 136 of the catering establishments were selected by a systematic sampling technique. Hence, every (3*n*)^th^, i.e., “408/3 = 136,” catering establishment was included in the sample where *n* ranges from 1 to 136. For food handlers from establishments, those who have greater than one food handler, at least one person who has a close contact with food (preparing foods) and food contact surfaces and equipment which was selected by a random sampling method and those who have only one food handler, he/she was directly involved in the research and keenly observed for the assessment of personal hygiene and hygienic practice and also interviewed to assess kitchen activity performance. Additionally, health status assessment and stool examination were also done [27].

### 2.5. Data Collection Method

#### 2.5.1. Structured Face-to-Face Interview

Data were collected by three well-trained persons through structured questionnaires to obtain information regarding age, sex, residence, family size, and occupation. Further, an in-depth interview was conducted to collect qualitative data. Key informants from selected managers and stakeholders were interviewed. Summary notes were taken and processed in the computer.

#### 2.5.2. Collection of the Stool Sample

Each participant was given a plastic container for sample collection and an applicator stick. About 5 g of fresh stool sample was collected from all study participants in a tight lead plastic container. The unique code (sex, age, and grade level) of the respondent was labeled on the submission of the stool sample. A portion of the stool was preserved in 10% formalin for helminthes, and for protozoans, it was preserved in 5% formalin. The stool specimens were transported in an ice box immediately to the Bule Hora general hospital for laboratory analysis [27].

#### 2.5.3. Direct Saline Thin Smear Wet Mount Microscopy

Direct stool examination was carried out according to the techniques described previously [3]. Briefly, two wet preparations of fresh stool from the same food handler were prepared with an amount of stool specimen of 0.25 mg emulsified with the formal saline on one end of a glass slide and Lugols iodine on the opposite end of the same slide. The two preparations were then covered with cover slips and examined under a light microscope for the presence of any parasites.

#### 2.5.4. Formal Ether Concentration Technique

The concentration technique was carried out using 3 g of fresh stool sample emulsified in 7 mL of formal saline as mentioned previously [3]. The resulting suspension was filtered through three layers of wet cotton gauze through a funnel in a centrifuge tube, and 3 mL of diethyl ether was added. The tube was centrifuged at 2500 rpm for 5 minutes, and the supernatant was poured off. Two wet preparations were made out of the sediment and covered with a cover slip. Finally, the slides were examined for the presence of parasites and type of parasites under a microscope [3].

### 2.6. Statistical Analysis of Data

The chi-square test was used to test for the significance level. The association between intestinal parasitic infection and sociodemographics, knowledge, and practices was calculated using the Poisson regression at 95% of confidence level. All statistical analyses were performed using SPSS version 20 [28].

## 3. Results

### 3.1. Demographic Characteristics of the Study Participants

A total of 136 participants working in 136 different catering establishments who met the inclusion criteria were recruited into this cross-sectional study.

#### 3.1.1. Distribution of Participants with Regard to Establishment Criteria

From dining rooms (49 (36.0%)), hotels (39 (25.8%)), bars and restaurants (19 (14.0%)), cafe and restaurants (12 (8.8%)), groceries and dining rooms (11 (8.1%)), cafe (7 (5.1%)), and cafeteria (3 (2.2%)) participants were sampled (Table 1). A significant difference was found in the distribution of study participants with regard to the catering establishments where they worked (*χ*^2^ = 14.000; df = 5; *P* = 0.001) (Table 1).

#### 3.1.2. Distribution of Participants with Regard to Gender

The total number of 46 (33.8%) males versus 90 (66.2%) females was studied. Consequently, there were significantly more females relative to male participants (*χ*^2^ = 8.389; df = 1; *P* = 0.001) (Table 1).

#### 3.1.3. Distribution of Participants with Regard to Age

The mean age of the participant was 24.73 (±3.226) years with a median of 25 years (range 20 to 37 years). The majority of the object (i.e., 116 (85.3%)) participants were aged between 21 and 30 years while the least 8 (5.9%) were aged from 31 to 37. A significant difference was noted in the distribution of study participants with regard to age (*χ*^2^ = 19.000; df = 3; *P* = 0.001) (Table 1).

#### 3.1.4. Distribution of Participants with Regard to Education Level

95 (69.9%) of the participants had a secondary level of education. The remaining 33 (24.3%) were having a tertiary level of education while the least (i.e., 8 (5.8%)) attended primary or basic education (Table 1). A significant difference was found in the distribution of study participants with regard to educational level (*χ*^2^ = 14.268; df = 3; *P* = 0.001).

#### 3.1.5. Distribution of Participants with Regard to Marital Status

93 (69.8%) of the study participants were found to be single. 39 (28.7%) of them were married while 4 (2.9%) of them were currently divorced. There was a significant difference observed in the distribution of study participants with regard to marital status (*χ*^2^ = 4.067; df = 3; *P* = 0.001) (Table 1).

#### 3.1.6. Distribution of Participants with Regard to Household Population Size

The mean number of the participant's household population was 2.51 (±0.827) persons with a median of 2 (range 0 to 3 persons). The majority (41 (30.1%)) of the participants were from a household with 1 to 3 occupants while 2 (1.5%) have no children. A significant difference was found in the distribution of study participants with regard to the household population (*χ*^2^ = 14.268; df = 2; *P* = 0.001) (Table 1).

### 3.2. Socioeconomic Characteristics of the Study Participants

#### 3.2.1. Distribution of Participants with Regard to Monthly Income

About 3 (2.2%) of the participants earned 10-25 USD, 59 (43.4%) has an income of 25 to 40 USD, 43 (31.6%) generate 40 to 50 USD, 9 (6.6%) have 50 to 65 USD of monthly income, and 22 (16.2%) have 65 USD and above as their monthly income. A significant difference was associated with the monthly income of study participants (*χ*^2^ = 29.617; df = 4; *P* = 0.001) (Table 2).

#### 3.2.2. Distribution of Participants with Regard to Housing Type

The majority (i.e., 58 (42.7%)) of the participants resided in a rental house while 55 (40.4%) live with the owners of the establishments, 21 (15.4%) are living with family, and 2 (1.5%) have their own house. A significant difference was observed in the distribution of study participants with regard to housing type (*χ*^2^ = 58.148; df = 2; *P* = 0.001) (Table 2).

#### 3.2.3. Distribution of Participants with Regard to the Source of Cooking Energy

The vast majority (i.e., 124 (91.2%)) of the study participants used firewood as a cooking source while 12 (8.8%) preferred electric energy for cooking purpose. A significant difference was found in the distribution of study participants in terms of their energy for cooking (*χ*^2^ = 9.356; df = 3; *P* = 0.001) (Table 2).

#### 3.2.4. Distribution of Participants with Regard to the Source of Household Lighting

119 (87.5%) of the participants used electricity as their light energy source. Other (i.e., 8 (5.9%)) used kerosene while 9 (6.6%) used solar energy as a source of lighting. A significant difference was recorded in the distribution of study participants with regard to the household lighting energy source (*χ*^2^ = 76.101; df = 2; *P* = 0.001) (Table 2).

### 3.3. Participants' Knowledge towards Intestinal Parasites

#### 3.3.1. Knowledge of Parasites

A maximum number of participants (i.e., 79 (68.1%)) were aware of intestinal parasites compared to 57 (41.9%) who had no knowledge about intestinal parasites (Table 3). A significant difference was found in the distribution of study participants with regard to their knowledge of intestinal parasitic infections (*χ*^2^ = 13.101; df = 3; *P* = 0.001).

#### 3.3.2. Need for Medical Examination

About 123 (90.4%) of the study participants were not aware about the purpose of medical examination when compared to the 13 (9.6%) remaining population. A significant difference was recorded for the distribution of study participants with regard to knowledge for medical examination (*χ*^2^ = 8.322; df = 1; *P* = 0.001). 13 (9.6%) of the participants were aware of the frequency of these medical examinations yearly in comparison to 123 (90.4%) participants who were not aware. Of those who were aware of the frequency of medical examination, 5 (38.5%) responded that they usually performed medical checkup twice per year and 8 (61.5%) responded that they used to carry out medical checkup routinely three times annually (Table 3).

#### 3.3.3. Awareness on the Legal Consequences for the Lack of Medical Examination

Nearly 123 (90.4%) of the participants were not aware of the legal consequences for not taking the regular medical examinations to their counterpart who were aware and said they have no information towards the consequence 13 (9.6%). A significant difference was seen towards the distribution of study participants with regard to knowledge on the legal consequences for the lack of medical examination (*χ*^2^ = 7.101; df = 1; *P* = 0.003) (Table 3).

### 3.4. Symptoms Associated with Intestinal Parasites

**Figure 2:**
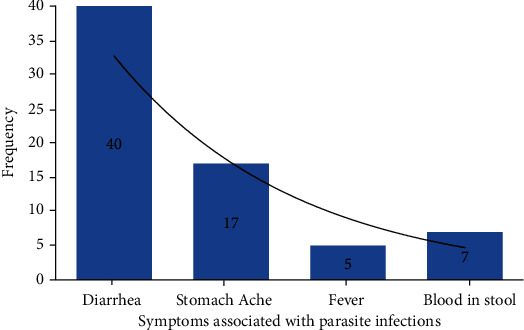
Respondents' knowledge towards signs and symptoms associated with intestinal parasites from Bule Hora Town, Ethiopia.

A significant difference was noted towards the study participants based on the knowledge of the types of intestinal parasites. From the survey, the researcher asked 79 (58.1%) participants who said they are aware of intestinal parasites along with signs and symptoms associated with intestinal parasite infection. The majority (40 (50.6%)) of respondents stated diarrhea followed by 17 (21.5%) who were aware of stomach ache. Others included 15 (19.0%) who said fever, and 7 (8.9%) stated the presence of blood in stool (Figure 2).

### 3.5. Practices of Participants regarding Intestinal Parasites

#### 3.5.1. Hand Washing Practices

As highlighted in Table 4, the majority (i.e., 117 (86.0%)) of study participants stated that they wash their hands regularly, 11 (8.1%) clean their hands sometimes, and 8 (5.9%) of them scrubbed their hands rarely. A significant difference was noted for participants towards their hand washing habit (*χ*^2^ = 62.067; df = 1; *P* = 0.001).

#### 3.5.2. Sanitation and Cleanliness

Nearly half (i.e., 66 (48.5%)) of study participants worked in catering establishments that had specific people employed to clean work places and toilets compared to 70 (51.5%) who did not have such kind of employees and facilities. There was a significant difference observed in the frequency of study participants based on the presence of specific people employed to clean work place and toilets (*χ*^2^ = 7.732; df = 1; *P* = 0.005) (Table 4).

#### 3.5.3. Personal Hygiene

The total number of 123 (90.4%) respondents stated that they cut their nails regularly than those of 13 (9.5%) who were habituated of trimming the nails rarely. No significant difference was seen in the frequency of study participants based on how regularly they cut their nails (*χ*^2^ = 62.914; df = 1; *P* = 0.05). The majority (i.e., 87 (63.9%)) of the participants acknowledged wearing of protective clothes during cooking in comparison to 49 (36.1%) who did not use such protection in kitchen. A significant difference was recorded in the frequency of study participants based on the practice of wearing protective clothes (*χ*^2^ = 39.141; df = 1; *P* = 0.001) (Table 4).

#### 3.5.4. Purpose of Hand Washing

**Figure 3:**
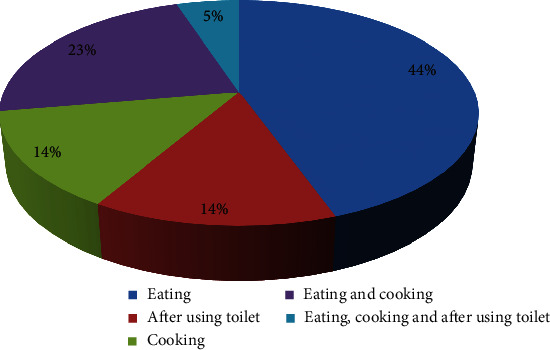
Participants' awareness towards the purpose of hand washing from Bule Hora Town, Ethiopia.

61 (44.8%) of study participants said that they used to do for eating purpose. 31 (22.8%) stated two purposes of hand washing, i.e., for eating and cooking. 19 (14.0%) used to wash their hands after using the toilet, 18 (13.9%) of them mentioned that they clean their hands for cooking purpose, and 7 (5.1%) said that they used to wash their hands for cooking, eating, and after using of toilet (Figure 3). A significant difference was achieved in the frequency of study participants based on the purpose of hand washing (*χ*^2^ = 30.550; df = 4; *P* = 0.001).

### 3.6. Laboratory Analysis

#### 3.6.1. Stool Appearance

**Figure 4:**
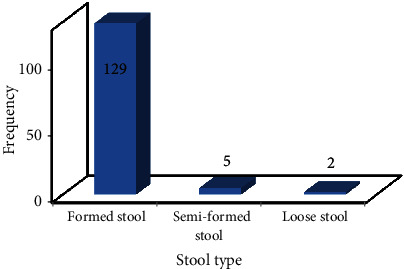
Stool type associated with study participants from Bule Hora Town, Ethiopia.

129 (94.9%) participants had formed stool, 5 (3.7%) had semiformed stool, and 2 (1.4%) had loose stool (Figure 4). A significant difference was recorded in the frequency of study participants based on the appearance of their stool samples (*P* = 0.001).

#### 3.6.2. Laboratory Diagnosis of Intestinal Parasites

**Figure 5:**
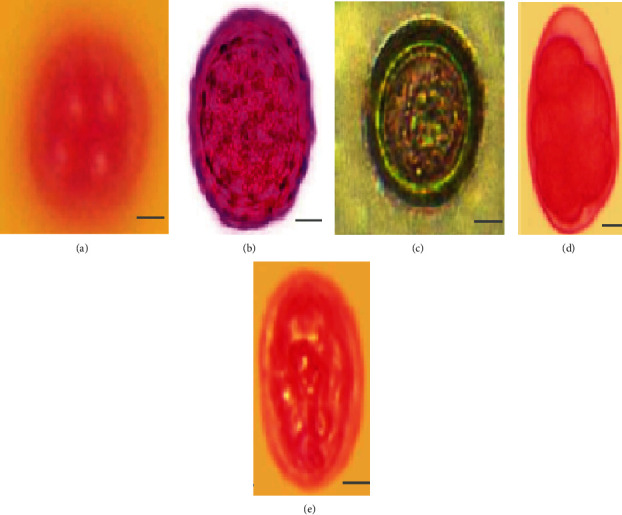
Diagnostic stages of intestinal parasites isolated from fresh stool samples of food handlers from Bule Hora Town, Ethiopia: (a) *Entamoeba histolytica* cyst; (b) *Ascaris lumbricoides* egg; (c) *Taenia saginata* egg; (d) hookworm egg; (e) *Giardia lamblia* cyst. Scale bar represents 100 *μ*m (magnification: ×400).

**Figure 6:**
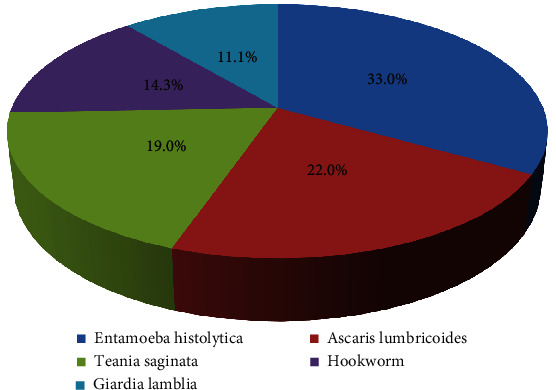
Frequency of distribution of intestinal parasitic infection among study participants from Bule Hora Town, Ethiopia.

63 (46.3%) were carriers of intestinal parasites while 73 (53.7%) had no cysts, trophozoites, larva, or eggs detected in their stool sample. Among 63 participants that were found to be positive for intestinal parasites, the majority (21 (33.3%)) were carrier of *Entamoeba histolytica*. Others included 14 (22.2%) carrying *Ascaris lumbricoides*, 12 (19.0%) participants had *Taenia saginata*, 9 (14.3%) respondents had hookworm, and 7 (11.1%) were associated with *Giardia lamblia* (Figures 5 and 6). A significant difference was observed in the prevalence of intestinal parasitic infection among study participants (*P* = 0.001).

### 3.7. Factors Associated with Intestinal Parasite Infections

#### 3.7.1. Demographic Characteristics

Bivariate and multivariate analyses of demographic factors revealed that none of participant's demographic variables such as work place, age, education level, marital status, and household population size were found to be associated with intestinal parasite infections (Table 5).

#### 3.7.2. Socioeconomic Factors

Participants whose household consumed vended or borehole water were more likely to be infected with intestinal parasite compared to those who had town municipal tap water facility. Generally, poor socioeconomic status had contributed to high prevalence of intestinal parasitic infection (Table 6).

#### 3.7.3. Knowledge-Related Factors

As shown in Table 7, none of the factors assessed such as knowledge of intestinal parasite, transmission of intestinal parasite, signs and symptoms associated with intestinal parasite, and past infection were found to be related to intestinal parasite infections.

#### 3.7.4. Practice-Related Factors

Table 8 demonstrates practice-related factors coherent with intestinal infection. Participants who stated washing of hands for the purposes of eating, after using toilet, and cooking or two of these reasons were less likely to get intestinal infection when compared to those who stated three different reasons for hand washing. On the other hand, the participants who responded that wearing of protective head gears were more likely to get intestinal parasitic infection compared to those who did not wear any head protective gear. Almost all hygiene practices analyzed were found to be associated with parasite infections significantly.

## 4. Discussion

Food handlers have potential of carrying a wide range of enteropathogens and have been implicated in the transmission of many infections to the public in the community and to patients in hospitals. Reports globally have emphasized the significance of food handlers with poor personal hygiene as a risk of transmission of parasitic and bacterial diseases [22]. There are currently 408 catering establishments in Bule Hora Town [23]. These catering establishments are not only visited by the locals but also attract high numbers of international tourists including dignitaries.

This study is among the first report on the prevalence and correlates of intestinal parasitic infection among food handlers within the Bule Hora Town clientele. The overall prevalence of intestinal parasite infections was 46.3%. The high prevalence of intestinal parasitic infections (46.3%) in this study among food handlers was in agreement with the findings of other studies conducted in Ethiopia like Addis Ababa (45.3%) [8], Yebu Town (44.1%) [29], Bahir Dar (41.1%) [15], and Nekemte Town (52.1%) [30] and in places apart from Ethiopia like Zulia State, Venezuela (48.7%) [31]; Minas Gerais, Brazil (47.1%) [32]; and Irbid, Jordan (48.0%) [33]. Higher rates in this study may be attributed to improper hygiene of food handlers. Higher prevalence of intestinal parasites was reported in Ethiopia from the Teda Health Center (62.3%) [34], East and West Gojjam (61.9%) [35], and elsewhere in Nigeria (97.0%) [36], Iran (74.0%) [37], and Anatolia, Turkey (52.2%) [38]. However, lower prevalence was reported in Sudan (29.4%) [39], Gaza Strip, Palestine (24.3%) [40], Turkey (8.8%) [41], Khuzestan, Southwest of Iran (7.7%) [42], North India (1.3 to 7%) [43], Thailand (10.3%) [44], and Chagni Town, Ethiopia (14.8%) [45]. This difference can be explained by research methodology difference, research time, sample size difference, epidemiological and environmental distribution difference, improved personal hygiene practices, environmental sanitation, and ignorance of health promotion practices.

In this study, the majority of parasitic infection (21 (33.3%)) was *E. histolytica* followed by 14 (22.2%) with *Ascaris lumbricoides*, 12 (19.0%) with *Taenia saginata*, 9 (14.3%) with hookworm, and 7 (11.1%) with *Giardia lamblia* (Figures 5 and 6). Similar parasitic dominance of *E. histolytica* (56.6%), *Ascaris lumbricoides* (26.4%), and *G. lamblia* (1.6%) was reported in Ethiopia from Nekemte Town [30] (*E. histolytica* (56.6%), *Ascaris lumbricoides* (26.4%), and *G. lamblia* (1.6%)*—*Addis Ababa [8], Bahir Dar [46], Kenya [47], and Turkey [41]). Other studies have identified *G. lamblia* as the leading parasite followed by other parasites such as those in Bahir Dar, Ethiopia [15], and in Iran [42]. Kamau et al. [3] in Kenya reported *Giardia* parasite as one of 6 common types of parasites among members of restaurant staff.

This study did not find any relationship between intestinal parasitic infection and participant's residency, age, education level, marital status, income, and household population size (Table 5). However, most of the food handlers in this study were female and young in age (below 30 years) and had lower secondary level education and low monthly income below USD 50. These characteristics of our food handlers are similar to a larger extent in other settings. A study in Ethiopia by Mama and Alemu [48] showed that most of the food handlers were females and young adults and had low educational levels, which is in line with studies from different parts of the world [8, 15, 49]. However, no significant difference between male (23 (50.0%)) and female (40 (44.4%)) in terms of intestinal parasitic infection was noted. This is in contradictory to the study of Mama and Alemu [48] that reported higher proportion of infected female food handlers (22.6%) with intestinal parasites relative to infected male food handlers (12.0%). This can be due to the fact that women are much more involved in kitchen work than men. Most of the males participate in the delivery of the already prepared food, while women are those who go bare footed during the preparation of the food, as well as those who do the washing of vegetables and fruits mainly in the kitchen.

Concerning the relation of the age group and parasitic infection, cumulatively although not significant, the study revealed relatively a higher infection rate in the age group younger than 30 years. No significant difference was found in the distribution of parasitic infection among all age groups which showed that there is equal exposure to the infection and suggests an effect of environmental conditions on the infection. This outcome is similar to various reports in India, Ethiopia, and other regions of the world [48–50]. Another factor, i.e., monthly income was the top contributor to intestinal parasitic infection in this study, consumption of vended water or borehole water was highly associated with intestinal parasitic infection. It is particularly not surprising for this association, as most Ethiopians has low monthly income status vended water or borehole in most parts which are never carefully handled according to the WHO standards including proper treatment and protection from external contamination. Studies have shown that the environmental route of transmission is important for many protozoan and helminthes parasites, with water, soil, and food being particularly significant. Both parasites have the potential for producing large numbers of transmissive stages and their environmental robustness, being able to survive in moist microclimates for prolonged periods of time, pose a persistent threat to public and veterinary health [51]. Drinking water has been observed as a major source of microbial pathogens in developing regions [52]. Generally, source of water have been linked to the socioeconomic status of the population with many reports showing a higher prevalence of intestinal parasitic infection more commonly in rural areas and in lower socioeconomic strata [49]. These reports have attributed this to probably an inability to afford and maintain food and water cleanliness.

Results of our study also revealed a significant overall relationship between food handler‘s sanitation and hygiene and intestinal parasitic infection. Food handlers hand washing reasons for the purposes of eating, after using the toilet, cooking or two of these reasons were less likely to get intestinal infections. On the other hand, food handlers who wore general protective headgears were more likely to get intestinal parasitic infections. Other studies have also reported several environmental and behavioral variables significantly contributing to intestinal parasite infection [21]. Like in this study, reduced hand washing with soap prior to eating, after using the toilet, or in both situations, and contact with soil, significantly increased the risk of intestinal parasitic infection [7, 21]. Other studies have also shown hand washing practice to be a determinant for intestinal parasitic infection among food handlers [15, 53]. Improper hand washing before handling food is one obvious route for dissemination of infections. Parasite eggs in the soil can be transmitted to vegetables, then on to hands and hence directly into the mouth [54], or ingested by eating raw vegetables [55]. Examination of finger nail contents of food handlers for ova or parasites is one way of indicating the possible contamination of food [56, 57].

Notably, this study did not report any association towards the respondent's knowledge-related factors (knowledge of intestinal parasite, transmission of intestinal parasite, problems associated with intestinal parasite, and past infection) to intestinal infection. Based on the participant's responses, it can be concluded that generally intestinal parasitic literacy level was higher in this population. A study in Southeast Asia showed that food handlers had relatively less knowledge about these infections; thus, there are more infections in those regions [58], while the infection level is less in developed countries like Italy [59]. As reported by Balarak et al. [60], literacy level reduces the number of positive samples; in other words, there is a significant relationship between level of education and degree of parasitic infection. It could be interpreted that if the literacy rate increased, then awareness about parasitic infections will also increase. Therefore, the lower need for health advice and better compliance with sanitary regulations will be achieved, as noted in other studies [61].

## 5. Conclusion

The prevalence of intestinal parasitic infection was high (46.3%) among food handlers showing high prevalence and thus consistent to other previous studies in Ethiopia and elsewhere. Most of the food handlers just as in other regions were infected with *E. histolytica* while hookworm and *G. lamblia* were the least common. Low socioeconomic status indicators such as utilization of vended or bore hole water and general poor personal hygiene were the major risk factors for the high prevalence of intestinal parasitic infection among food handlers found in our report. Therefore, mitigating steps such as enforcement of systems that promote improvement of personal- and facility-level hygiene, more public training, and wider enforcement of medical certification policy are vital to possibly reduce the risk of parasite infections.

## Figures and Tables

**Table 1:** Demographic characteristics of the study participants from Bule Hora Town, Ethiopia.

Variable	Unit	Number (*N*)	%	*χ* ^2^	Degree of freedom	*P* value
Catering establishments	Hotels	39	25.8	14.000	5	0.001
	Bar and restaurants	19	14.0			
	Cafe and restaurants	12	8.8			
	Dining rooms	49	36.0			
	Cafes	7	5.1			
	Food and groceries	11	8.1			
	Cafeteria	3	2.2			
Gender	Male	46	33.8	8.389	1	0.001
	Female	90	66.2			
Age (years)	15-20	12	8.8	19.000	3	0.001
	21-30	116	85.3			
	31-40	8	5.9			
Educational level	Primary	8	5.9	14.268	3	0.001
	Secondary	95	69.9			
	Tertiary	33	24.2			
Marital status	Single	93	68.4	4.067	3	0.001
	Married	39	28.7			
	Divorced	4	2.9			
Household population size	1	24	17.6	14.268	2	0.001
	2-3	17	12.5			
	None	2	1.5			

^∗^*χ*^2^ = chi-square; *P* = level of significance; *P* ≤ 0.05 indicates that the relationship is significant.

**Table 2:** Sociodemographic characteristics of the study participants from Bule Hora Town, Ethiopia.

Variable	Unit	Number (*N*)	%	*χ* ^2^	Degree of freedom	*P* value
Monthly income (USD)	10-25	3	2.2	29.617	4	0.001
	25-40	59	43.4			
	40-50	43	31.6			
	50-65	9	6.6			
	65 and above	22	16.2			
Housing type	Rental house	58	42.7	58.148	2	0.001
	Own house	2	1.5			
	Live with family	21	15.4			
	Live with the owner	55	40.4			
Cooking energy source	Firewood	124	91.2	9.356	3	0.001
	Electricity	12	8.8			
	Kerosene	8	5.9			
Lighting energy source	Solar	9	6.6	76.101	2	0.001
	Electricity	119	87.5			
	Kerosene	8	5.9			

^∗^*χ*^2^ = chi-square; *P* = level of significance; *P* ≤ 0.05 indicates that the relationship is significant.

**Table 3:** Knowledge of the study participants towards intestinal parasites from Bule Hora Town, Ethiopia.

Variable	Unit	Number (*N*)	%	*χ* ^2^	Degree of freedom	*P* value
Intestinal parasite awareness	Yes	79	58.1	13.101	3	0.001
	No	57	41.9			
Medical exam frequency	Yes	13	9.6	3.879	1	0.049
	No	123	90.4			
No. of times exam preferred	Twice per year	5	38.5	—	—	—
	Thrice per year	8	61.5			
Legal consequences towards medical exam	Know	7	53.8	7.101	1	0.003
	Do not know	6	46.2			

^∗^*χ*^2^ = chi-square; *P* = level of significance; *P* ≤ 0.05 indicates that the relationship is significant.

**Table 4:** Hygiene practices of the study participants from Bule Hora Town, Ethiopia.

Variable	Unit	Number (*N*)	%	*χ* ^2^	Degree of freedom	*P* value
Practice of hand washing	Always	117	86.0	62.067	1	0.001
	Sometimes	11	8.1			
	Rarely	8	5.9			
Facility of washing toilet	Yes	66	48.5	7.732	1	0.005
	No	70	51.5			
Nail trimming practice	Yes	123	90.4	62.914	1	0.05
	No	13	9.5			
Wearing of protective clothes	Yes	92	67.6	39.141	1	0.001
	No	44	32.4			

^∗^*χ*^2^ = chi-square; *P* = level of significance; *P* ≤ 0.05 indicates that the relationship is significant.

**Table 5:** Demographic characteristics associated with parasite infection of the study participants from Bule Hora Town, Ethiopia.

Variable	Unit	Number (*N*)	Frequency of parasite infection	% of parasite infection	*χ* ^2^	*P* value
Catering establishments	Hotels	39	16	45.7	35.436	0.227
	Bar and restaurants	19	8	42.1		
	Cafe and restaurants	12	8	66.7		
	Dining rooms	49	24	49.0		
	Cafes	7	5	45.5		
	Food and groceries	11	1	14.3		
	Cafeteria	3	1	33.3		
Gender	Male	46	23	50.0	8.356	0.138
	Female	90	40	44.4		
Age (years)	15-20	12	07	58.3	10.644	0.386
	21-30	116	52	44.8		
	31-40	8	4	50.0		
Educational level	Primary	8	1	12.5	7.700	0.658
	Secondary	95	48	50.5		
	Tertiary	33	14	42.4		
Marital status	Single	93	50	50.0	28.860	0.236
	Married	39	11	28.2		
	Divorced	4	2	50.0		
Household population size	1	24	12	50.0	12.193	0.543
	2-3	17	6	35.2		
	None	2	00	00.0		

^∗^*χ*^2^ = chi-square; *P* = level of significance; *P* ≤ 0.05 indicates that the relationship is significant.

**Table 6:** Sociodemographic characteristics associated with parasite infections of the study participants from Bule Hora Town, Ethiopia.

Variable	Unit	Number (*N*)	Frequency of parasite infection	% of parasite infection	*χ* ^2^	*P* value
Monthly income (USD)	10-25	3	3	100	28.955	0.009
	25-40	59	29	49.1		
	40-50	43	21	48.8		
	50-65	9	4	44.4		
	65 and above	22	6	27.2		
Housing type	Rental house	58	23	39.7	15.412	0.042
	Own house	2	2	100		
	Live with family	21	10	43.5		
	Live with the owner	55	28	50.9		
Source of drinking water	Municipal tap water	128	58	45.3	8.779	0.018
	Borehole water	8	5	62.5		
Cooking energy source	Firewood	124	54	43.5	14.593	0.482
	Electricity	12	9	75.0		
	Kerosene	8	3	37.5		
Lighting energy source	Solar	9	5	43.7	17.045	0.073
	Electricity	119	52	75.0		
	Kerosene	8	6	55.6		

^∗^*χ*^2^ = chi-square; *P* = level of significance; *P* ≤ 0.05 indicates that the relationship is significant.

**Table 7:** Knowledge-related factors associated with parasite infections of respondents from Bule Hora Town, Ethiopia.

Variable	Unit	Number (*N*)	Frequency of parasite infections	% of parasite infections	*χ* ^2^	*P* value
Intestinal parasite awareness	Yes	79	31	39.2	6.297	0.278
	No	57	32	56.1		
Transmission of intestinal parasites	Ingestion	46	21	45.6	20.389	0.434
	Person to person	16	5	31.3		
	Skin penetration	4	2	50.0		
	Inhalation	1	1	100		
	Do not know	12	3	25.0		
Medical exam frequency	Yes	13	7	53.8	4.321	0.504
	No	123	56	45.5		
No. of times exam preferred	Twice per year	5	2	40.0	6.663	0.155
	Thrice per year	8	5	62.5		
Legal consequences towards medical exam	Know	7	4	57.1	4.280	0.369
	Do not know	6	3	50.0		
Symptoms associated with parasite infections	Diarrhea	40	12	30.0	15.360	0.933
	Stomach pain	17	3	37.5		
	Fever	15	3	20.0		
	Blood in stool	7	0	00.0		

^∗^*χ*^2^ = chi-square; *P* = level of significance; *P* ≤ 0.05 indicates that the relationship is significant.

**Table 8:** Relation of parasitic infections with hygiene practices of the study participants from Bule Hora Town, Ethiopia.

Variable	Unit	Number (*N*)	Frequency of parasite infections	% of parasite infections	*χ* ^2^	*P* value
Practice of hand washing	Always	117	49	41.8	22.756	0.001
	Sometimes	11	9	81.8		
	Rarely	8	5	62.1		
Hand washing purpose	Eating	61	32	52.5	17.783	0.032
	After toilet	19	11	57.9		
	Cooking	18	7	38.8		
	Eating and cooking	31	11	35.5		
	All of these	7	2	28.5		
Facility of washing toilet	Yes	66	21	31.8	4.042	0.050
	No	70	42	60.0		
Nail trimming practice	Yes	113	54	43.9	22.003	0.040
	No	23	9	69.2		
Wearing of protective clothes	Yes	87	48	55.2	29.468	0.034
	No	49	15	30.6		

^∗^*χ*^2^ = chi-square; *P* = level of significance; *P* ≤ 0.05 indicates that the relationship is significant.

## Data Availability

Raw data can be obtained from the corresponding author upon kind request.
